# 

**Published:** 1889-06

**Authors:** 


### CONTENTS.

***MISCELLANEOUS.*** PAGE

On Gonorrhœa in its Constitutional Aspects. Dr. J. Compton Burnett, . . . 217
On Gonorrhœa in its Constitutional Aspects. W. M. J., . . . 228
Proceedings of Boston Organon Society, . . . 229
Proceedings of Oneida County Medical Society, . . . 232
Notes from Past Meetings of the Lippe Society. Dr. George H. Clark, . . . 234
Bryonia—Some Notes. Dr. H. E. Potter, . . . 239
A Peculiar Case. Dr. H. E. Potter, . . . 240
A Lilium Tigrinum Case. Dr. E. W. Berridge, . . . 241
Lilium Tigrinum and Prolapsus Uteri. Dr. T. G. Roberts, . . . 242
Action of Alumina in Infantile Paralysis. Dr. E. W. Berridge, . . . 243
Therapeutic Observations upon Carbo Vegetabilis. Dr. C. Hering, 244
Mental Derangements. Dr. G. W. Sherbino, . . . 246
Some Clinical Cases. Dr. R. M. Theobald, . . . 248
Clinical Cases. Dr. Clarence N. Payne, . . . 250
More Croup. Dr. W. S. Gee, . . . 250
Lac Vaccinum. Dr. S. Swan, . . . 252
Fragmentary Provings. Dr. R. M. Theobald, . . . 253
A Proving of Ustilago Maidis. Dr. S. Swan, . . . 253
Verification of a Lachesis Symptom. Dr. J. E. Russell, . . . 254
Some Practical Notes. Dr. H. C. Morrow, . . . 254
In Memoriam—
George F. Foote, . . . 255
Dr. G. Felix Matthes, . . . 256
A New Remedy, and a New Indication for an Old One. Dr. R. B. Johnstone, . . . 257
A Homœopathic Hospital in Italy, . . . 257
What are the Remedies? . . . 258
Sciatica. Dr. B. Simmons, . . . 258
A Verification. E. A. Ballard, . . . 259
Extract from the Address of Dr. George Wigg, . . . 259

***BOOK NOTICES.***

Lectures upon Diseases of the Heart, by E. H. Hale, M. D., . . . 263
Electricity and the Methods of its Employment, etc., by Plym S. Hayes, M. D., . . . 263
Electro-Therapeutics, by William Harvey King, M. D., . . . 263
The Cincinnati Enquirer, . . . 264

***NOTES AND NOTICES.***

Erratum—Removals—The Annals of Surgery—For Sale, . . . 264

